# Smart forage selection could significantly improve soil health in the tropics

**DOI:** 10.1016/j.scitotenv.2019.06.152

**Published:** 2019-10-20

**Authors:** C.A. Horrocks, J. Arango, A. Arevalo, J. Nuñez, J.A. Cardoso, J.A.J. Dungait

**Affiliations:** a Department of Sustainable Soil and Grassland Systems, Rothamsted Research, North Wyke, Okehampton, Devon EX20 2SB, UK; b International Center for Tropical agriculture (CIAT), Km 17, Recta Cali–Palmira, CP 763537, Apartado Aéreo 6713, Cali, Colombia

## Abstract

The use of tropical grasslands to graze livestock is of high economic importance. Declining grassland soil health leads to reduced sustainability of livestock systems. There are high levels of phenotypic diversity amongst tropical forage grasses. We hypothesise that this variation could lead to significant differences in soil health and that selection of forage cultivars to improve soil health could improve the sustainability of livestock production. We measured and compared key soil health metrics (soil organic carbon (SOC) concentration and sugar / alkane composition, aggregate stability, friability, litter decomposition rates, microbial community composition) under four tropical forage varieties (Brachiaria hybrid cv Mulato (BhMulato), *B. humidicola* cv Tully (CIAT679; Bh679), *B. humidicola* cv CIAT16888 (Bh16888), and *Panicum maximum* CIAT 6962 (Pmax)) and a bare soil control, there was a significant difference in soil aggregate stability, friability and SOC concentration between the forage varieties with soil under Bh679 and Bh16888 tending to have greater aggregate stability, friability and SOC concentrations compared to the soil under BhMulato and Pmax. We identified significant spatial variation in soils under BhMulato and Pmax due to their tussock forming growth habit; when compared to soil from adjacent to the tussocks, soil from the gaps between tussocks had significantly reduced aggregate stability under both species, significantly reduced friability under Pmax and significantly reduced SOC under BhMulato. We found limited impact of forage variety on soil microbial community composition, litter decomposition rates or soil alkane and sugar concentrations.

## Highlights

• Improved soil health was observed under certain *Brachiaria* species.
• Aggregate stability and friability in particular were improved under *B.humidicola*.
• Improved soil physical properties correlated with increased soil organic matter.
• Forage growth habit affected spatial variation in soil properties.
• Limited impact of forage variety was observed on soil biological properties.

## Graphical abstract

**Figure:**
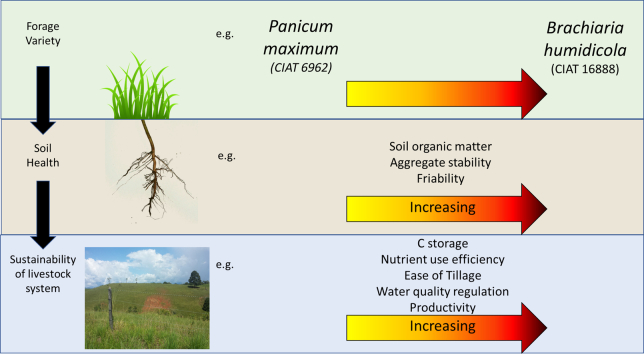

## 1 Introduction

Forage based livestock production systems represent about 70% of agricultural land in the tropics (Rao et al., 2011). Grassland savannah covers >250 million ha of South America, with over 50% of this area used for grazing beef cattle. In Brazil alone over 80 million ha are planted with tropical forages native to Africa from the genus *Brachiaria* (De Oliveira et al., 2004). The use of ‘Climate smart’ *Brachiaria* cultivars that have been selected for better adaptation to biotic and abiotic stress, in particular climatic stress, is being encouraged (Rao et al., 2011; Moreta et al., 2014) As well as being of high economic importance in tropical South America, *Brachiaria* grasses could play a key role in increasing agricultural sustainability in their native Africa through their use in the push-pull system (Djikeng et al., 2014). As part of the push-pull system *Brachiaria* cvs. Mulato and Mulato-II are used as ‘trap’ plants to increase maize yield by (Midega et al., 2015) ‘trapping’ stem borer moths and the parasitic plant Striga (*Striga hermonthica*), whilst providing a high-quality forage even under drought stress (Maass et al., 2015).

Concerted collection of *Brachiaria* germplasm in their native East Africa has resulted in the development of extensive ex-situ collections, holding over 980 different accessions and 33 known species expressing high levels of morphological and physiological diversity (Keller-Grein et al., 1996; Guenni et al., 2002), hence there is great potential for selective breeding to develop improved cultivars by selection of desirable agronomic traits combining productivity and soil health.

Healthy agricultural soils have been defined as those which provide and continue to provide both an adequate level of agricultural productivity as well as other ecosystem services on which humans depend (Kibblewhite et al., 2008), such as the regulation of efficient nutrient cycles, support of biodiversity, reduction of greenhouse gas emissions, regulation of hydrological cycles and maintenance of water quality (Horrocks et al.,2014). Globally declining soil health in agricultural systems has led to a loss of organic matter, erosion, increased compaction, decreased diversity of soil organisms, reduced nutrient cycling efficiency, reduced drought tolerance and reduced yield /quality of outputs (Kibblewhite et al., 2008). Agricultural land management practices have a major influence on soil health and the need to develop land management strategies aimed at improving soil health has been identified as a global priority for sustainable development (Doran, 2002). Determining the state of soil health requires the measurement of multiple soil properties, (Kibblewhite et al., 2008) including physical, chemical and biological properties of soil (Parr et al., 1992). The use of more scientifically and technically demanding soil health metrics should be combined with the development of in-field assessment techniques that can be used to augment the existing understanding of soils held by farmers with a scientifically ratified soil health assessment protocol (Romig et al., 1995).

Soils under well managed *Brachiaria* pastures have shown qualities indicting good soil health, such as efficient nutrient use, low susceptibility to erosion, enhanced biological activity, good soil structure and large soil organic matter concentrations (Boddey et al., 1996). However, when Brachiaria pastures are not well managed they are susceptible to degradation (Boddey et al., 2004); over 25 million ha of *Brachiaria* pasture in Brazil are classified as degraded (De Oliveira et al., 2004).

Plant traits shown to affect soil health indicators include the quantity and composition of root exudates (Chaparro et al., 2012), root morphology (Miller and Jastrow, 1990), productivity (Zak et al., 2003) and litter quality (Boddey et al., 2004). One important trait identified in *Brachiaria* is their capacity to reduce soil nitrification through the secretion of specific root exudates (i.e. brachialactone), known as biological nitrification inhibition (BNI) (Subbarao et al., 2009). BNI in *Brachiaria* has been the focus of much research interest for its potential to improve nitrogen use efficiency of a subsequent crop (Karwat et al., 2017), reduce emissions of the greenhouse gas nitrous oxide (Byrnes et al., 2017) and reduce nitrogen leaching (Subbarao et al., 2012). Significant Intraspecific differences have been observed in the BNI activity (Nuñez et al., 2018) and other adaptations to biotic and abiotic stress (Rao et al., 2011; Moreta et al., 2014) amongst *Brachiaria,* which could in turn lead to contrasting impacts on soil health.

In this study we take the novel approach of comparing the impact on soil health of potential new and established tropical forage cultivars, including members of the commercially dominant *Brachiaria* genus. We assess multiple indicators of soil health under established field plots, going beyond the traditional focus on biological nitrification inhibition (BNI), productivity and drought / disease resistance to maximise our understanding of the impacts of forage identity on a range of soil properties that are essential to ecosystem service provision and sustainable agriculture. We include low tech methods that could be utilised by land managers in the fields to enable self-assessment of soil health and could prove a vital tool in the future of sustainable global food production.

We hypothesise that the physiological and morphological variability amongst *Brachiaria* grasses can be exploited to select for cultivars that have a positive impact on a range of soil health indicators and thus decrease the risk of pasture degradation. The importance of soil-plant-microbe interactions in the rhizosphere for plant health, could also lead to a correlation between improved soil health and improved forage quality and yield, leading to health benefits and increased profits for farmers (Jeffries et al., 2003; Chaparro et al., 2012), thus improving the economic, environmental and social sustainability of livestock production in the tropics.

In this study we aimed to compare the impact of different tropical forage cultivars on soil health and assess the potential to use low-tech methods of measuring soil health in the field. Using forage varieties developed at the International Centre for Tropical Agriculture (CIAT), our objectives were to measure a range of chemical (total organic carbon concentration, organic matter composition), biological (abundance and diversity of key microbial groups using genetic markers, potential organic matter decomposition rate) and physical (bulk density, aggregate stability and friability) properties of soil. By using a range of low-tech methods alongside those requiring more specialist equipment and skills, we planned to identify any significant differences in soil properties under the different forages and look for correlations between variables to identify any suitable low-tech methods for comparing soil health in the field.

## 2 Materials and methods

### 2.1 Study site

**Fig. 1:**
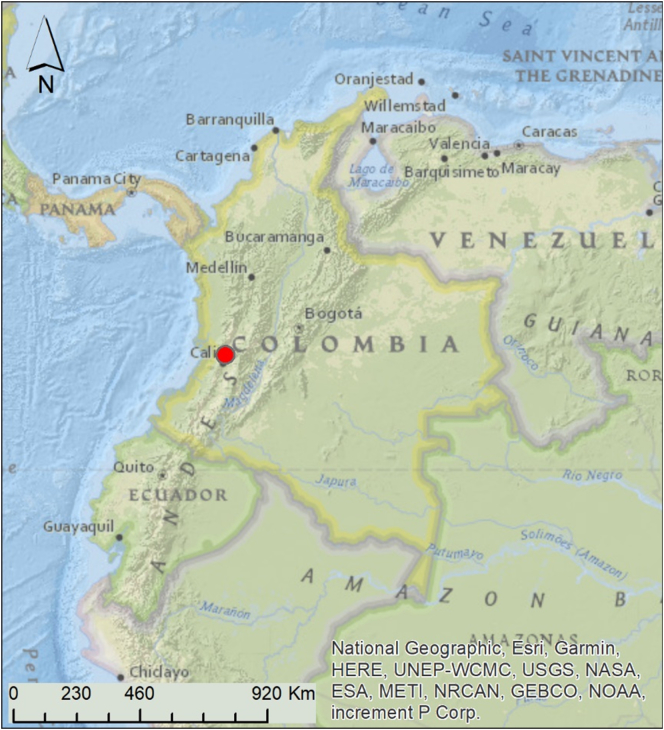
Map of Colombia showing location of CIAT, Palmira Valle del Cauca department.

We assessed soil health under an existing tropical forage field trial established in 2006 at CIAT in Palmira, Valle del Cauca Colombia (3^0^ 30′7″N 76^0^21′22″W, Fig. 1). The study site was a flat area 965 m above sea level. The soil was a Vertisol (Typic Pellustert; IGAC, 1980) with a silty clay loam texture (40–60% clay), the parent material was fluvial in origin from the erosion of igneous rock from the Andes (Howler, 1986). The study site falls within the tropical dry forest climatic region and has a mean annual rainfall of 894 mm and a mean annual temperature of 24 °C (Byrnes et al., 2017).

### 2.2 Forages

For this study, we selected perennial C4 tropical forage grasses with different growth habits and important agronomic attributes to identify their impact on soil health indicators. Three Brachiaria grasses were selected: *Brachiaria* hybrid Mulato I, selected for drought tolerance, high quality forage, spital bug resistance and high productivity in the tropics (Argel et al., 2007); *Brachiaria humidicola* CIAT 679, selected for high productivity, drought resistance, nutritional value and BNI potential (Byrnes et al., 2017; Subbarao et al., 2009; Guenni et al., 2002) and *Brachiaria humidicola* CIAT 16888, selected for tolerance to nutrient poor soils and high BNI activity (Arango et al., 2014; Nuñez et al., 2018). A member of the genus *Panicum,* grown widely throughout the tropics, was also included in the study plots, *Panicum maximum* (CIAT 6962), selected for high productivity when supplied with sufficient nutrients and water (Aganga and Tshwenyane, 2004).

### 2.3 Experimental design and sampling regime

**Fig. 2:**
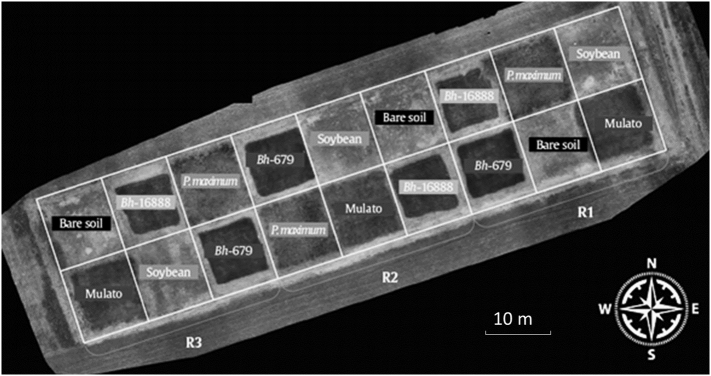
Aerial photo showing layout of field plots at CIAT Palmira. Includes three blocks (R1-R3) each containing one replicate plot of each of four tropical forage varieties including; *Brachiaria hybrid cv* Mulato (Mulato)*, B. humidicola cv* Tully (CIAT679; Bh-679*), B. humidicola cv CIAT16888* (Bh-16,888)*,* and *Panicum maximum* CIAT 6962 *(P.maximum),* a bare soil control was also included in the trial (Bare soil), the Soybean plots were not used in the present study.

The trial consisted of three replicate 10 m × 10 m plots in a randomized block design of each of four tropical forages; *Brachiaria hybrid cv* Mulato (BhMulato)*, B. humidicola cv* Tully (CIAT679; Bh679*), B. humidicola cv CIAT16888* (Bh16888)*,* and *Panicum maximum* CIAT 6962 *(*Pmax*),* a bare soil control was also included in the trial, and maintained by weeding and application of glufosinate-ammonium herbicide (Fig. 2). Samples were collected from the field plots in February 2016 and again in January 2017. In 2016 soils were sampled by collecting three intact 10 × 10 × 10 cm soil cubes from the top 10 cm of the soil profile in each of the bare soil, Bh679 and Bh16888 blocks, with care taken not to disturb the soil structure, these cores were then combined to give a single composite sample per plot prior to analysis. Whilst the Bh679 and Bh16888 plots had 100% grass cover, the tufted growth habit of BhMulato and Pmax resulted in large distances between tussocks within these plots, mean (n = 3) % ground cover by grass of 55.4 ± 2 and 68.2 ± 2.3% respectively (Supplementary Table 1), thus six soil samples were collected from each of these blocks, three samples were collected from adjacent to each tussock and three were collected from a gap between tussocks (defined as an area of soil > 10 cm from the nearest tussock). The sampling locations within each block were randomly selected by assuming each block was divided into 10 × 10 cm squares, which were chosen via random generation to select the x and y coordinates of the bottom left hand corner of the square to be sampled. In 2017 the sampling method above was repeated but this time, to ensure any intra-plot variability was more fully accounted for in the composite sample from each plot, five samples were collected from each bare soil, Bh679 and Bh16888 plot, and ten from each Pmax and BhMulato plot, five from adjacent to a tussock and five from a gap between tussocks. Soil analyses were carried out as detailed below.

### 2.4 Soil analyses

#### 2.4.1 Soil organic carbon concentration

Soil organic carbon (SOC) concentrations were determined for soil samples collected in 2016 and 2017. Samples were finely ground with a mechanical ball mill then left for 16 h in 1 M Hydrochloric acid to remove any inorganic C, the samples were then rinsed three times with ultrapure water to remove any acid and dried at 105 °C. Approximately 15 mg of sample was weighed out into a foil capsule and a Carlo Erba NA 1500 analyser (Carlo Erba, Val-de-Reuil, France) was used to obtain the % elemental mass of SOC.

#### 2.4.2 Extraction and quantification of soil sugars

We determined the concentration of the major hexose (glucose, galactose, mannose) and pentose (arabinose, xylose, rhamnose) sugar moieties in the soil samples collected in 2016 and 2017 to determine any differences in sugar composition under the different forage varieties. Sugars were extracted from previously lipid extracted soil (see method for lipid extraction in alkane analysis), using the method described by Blakeney et al. (1983) and adapted by Docherty et al. (2001). The sugars were quantified on an Agilent 7890A GC (Agilent, Berkshire, UK) fitted with and FID detector and a VF-23 ms column (60 m × 0.32 mm i.d. × 0.15 μm film thickness; Agilent, Berkshire, UK) The oven temperature was programmed to hold at 50 °C for 1 min then increase at 20 °C min^−1^ to 200 °C then at 4 °C min^−1^ to 230 °C and hold for 7 min. Compounds were identified based on retention time compared to a known standard and concentrations calculated by comparison of peak area to that of an internal pentaerythritol standard. The hexose to pentose ratio was calculated as the concentrations of mannose + galactose: arabinose + xylose.

#### 2.4.3 Extraction and quantification of soil n-alkanes

Straight chained alkanes (n-alkanes) with chain length of 20 carbon atoms (C20) to 33 carbon atoms (C33) inclusive were quantified for the soils collected in 2017 only, total lipid extracts were obtained from 10 g of freeze dried soil using a method based on that described by Bull et al. (2000), n-alkanes were quantified using an Agilent 7890A GC (Agilent, Berkshire, UK) fitted with an FID detector and an Agilent HP-5 column (30 m × 320 μm i.d. x 0.25 μm film thickness), oven temperature was held at 40 °C for 1 min then increased at 20 °C min^−1^ to 130 °C then at 4 °C per minute to 300 °C and held for 10 min. Peaks were identified based on known retention times determined using standard mixes and quantification carried out by comparing peak area to that of an internal nonadecane standard, total n-alkane concentrations were calculated as the sum of C20-C33 n-alkanes inclusive.

#### 2.4.4 Aggregate stability

Wet aggregate stability (WSA) was determined for the soils in both years of the study. Soils composed of less stable aggregates will be more vulnerable to erosion and crusting during rainfall events. In this study a protocol was developed through adaptation of the ‘fast wetting’ method described by Le Bissonais (1996). The protocol was developed such that, with small adjustments, it could be easily carried out by farmers or researchers in the field without access to laboratory equipment and would indicate the relative risk of soil erosion or crusting during heavy rainfall following a drought. Fresh soil samples were sieved to obtain aggregates >2 mm and ≤ 5 mm diameter prior to analysis, these aggregates were dried at 40 °C to constant weight (48 h) and left to cool in a desiccator. Low-cost sieves were constructed by fixing a 10 cm^2^ piece of nylon mesh (mesh size 50 μm diameter; Plastok meshes and filtration Ltd., Birkenhead, UK) with an elastic band around the top of a plastic beaker from which the base had been cut. Approximately 10 g dried aggregates were weighed into a sieve and the sieve placed in a beaker of deionised water to 7 cm depth for 10 min prior to sieving. A manual sieving method was used, adapted from Elliott (1986) the sieve was manually lifted completely from the water (7 cm) and replaced in it then moved side to side three times. This process was repeated five times for each sample, the sieve and soil were dried at 40 °C to constant weight and left to cool in a desiccator. The remaining soil was then re-weighed, and the percentage of soil lost through the 50 μm mesh was calculated (SoilLoss; % dry mass), this value was inversely proportional to WSA.

#### 2.4.5 Aggregate friability

The friability of soil (*F*) can be defined as

‘The tendency of a mass of unconfined soil to disintegrate and crumble under applied stress into a particular size range of smaller fragments’ (Utomo and Dexter, 1981).

Friability has been found to be positively correlated with aggregate stability and SOC concentration (Watts and Dexter, 1998). To determine the strength of any such correlations in our soils we determined *F* for the samples collected in 2017 only, using an indirect tension test (Rogowski et al., 1968; Dexter, 1975). A simple loading frame apparatus based on a design by Dexter et al. (1988) was constructed to determine *F*. The apparatus consisted of two arms hinged together, with the upper arm free to rotate around a pivot, part way up each arm is a crusher plate, the upper and lower plates come together so that they are parallel when an aggregate is placed on the lower plate. The device is positioned over the edge of a bench, so a plastic bucket can be hooked on the end of the upper arm. Aggregates are placed between the crusher plates and observed as water is poured into the bucket, the mass of water needed to initiate a fracture in the aggregate was recorded. *F*_1_ was calculated by the coefficient of variation method as described by Watts and Dexter (1998). Five aggregates approximately 10 mm in diameter were selected from each soil sampling location, giving a total of 25 aggregates per plot. The aggregates were dried at 105 °C and stored in a desiccator. Each aggregate was placed in the crushing apparatus and water was poured slowly into the bucket, the force generated by the known mass of water was calculated using Eq. (1).

(1) $$P=\mathrm{Wg}\;\left(\frac{x_{1}}{x_{2}}\right)$$

where W = the mass of water, g = acceleration of gravity = 9.81 ms^−2^, x_1_ = is the distance from the bucket to the pivot = 54 cm, x_2_ = distance from the pivot to the crushing plates = 36 cm.

Aggregates were selected that were near spherical and calculations of *F*_1_ are based on assumed sphericity. The tensile strength of each aggregate was calculated using Eq. (2).

(2) $$Y=0.576\frac{P}{D^{2}}$$

where *Y* = the tensile strength of an aggregate, *P* = force required to trigger fracture (from Eq. (1)) and *D* = the aggregate diameter. The value for *D* was determined for each aggregate by taking the mean length of the *x*, *y* and z axes.

The friability index (*F*_1_) for the soil at each sampling location was determined using Eq. (3),

(3) $$F_{1}=\frac{\sigma_{Y}}{\bar{Y}}\pm \frac{\sigma_{Y}}{\bar{Y}\sqrt{2n}}$$

where *σ*_Y_ = the standard deviation for the values of *Y* (calculated for the five aggregates from each sampling location). $\bar{Y}$ = the mean (n = 5) values of *Y* calculated for the aggregates from each location, n *=* the number of replicate aggregates from each sampling location = 5 replicates.

The greater the value of *F* the greater the friability of the soil.

#### 2.4.6 Soil DNA extraction and QPCR of target genes

Total soil DNA was extracted and quantified from soils collected in February 2016 only. The soil from each sampling location was crushed and homogenized and the three samples from each plot were combined to give a composite sample that was immediately frozen at −80 °C for one week. The frozen soil samples were freeze-dried and DNA was obtained from 100 mg of dry soil using the MP BIO kit, with modification during the washing step to reduce the co-extraction of PCR inhibitors such as humic acids (Nuñez et al., 2018). The total bacteria and archaea abundance was estimated using 16S genes. The 16S bacterial primers were 338F (ACTCCTACGGGAGGCAGCAG) and 518R (ATTACCGCGGCTGCTGG). For archaea, the primers were Arch109f (ACKGCCAGTAACACGT) andArch912r (CTCCCCCGCCAATTCCTTTA), qPCR conditions were applied according to Muema et al. (2016). The size of the fungal community was determined through quantification of the fungal 18S rRNA genes using the primers FF390 and FR1 (Vanio and Hantula, 2000). PCR runs for fungal 18S gene were performed on a MX3000p (Stratagene) starting with an initial denaturation at 95 °C for 3 min, and 45 cycles of denaturation at 94 °C for 30 s, annealing at 50 °C for 30 and extension at 70 °C for 1 min followed by a melting curve.

#### 2.4.7 Potential organic matter decomposition – Teabag test

We measured the potential for organic matter decomposition in each of the study plots in 2017 only, using a protocol based on the teabag index developed by Keuskamp et al. (2013). The teabag index uses commercially available tea as a substitute for litter bags such that a standardized test can be performed easily by people outside of the research community. In our study two teabags containing more readily decomposed green tea (Lipton®), two tea bags containing less decomposable roobois tea (Lipton®) and two teabags containing a rooibos tea from a brand more readily available in Colombia were buried at 8 cm depth in the soil in each of the study plots. Each teabag was weighed prior to burial and the mass of tea calculated by subtracting the mean (n = 5) mass of the bag from dismantled teabags from the total mass of each teabag prior to burial. One of each type of teabag was recovered from each plot after 30 days. The remaining tea was separated from the bag and any soil particles and dried at 50 °C, the mass of tea remaining was recorded and the proportional loss in tea mass was calculated for each tea type after 30 days. The litter decomposition rate (*k*)calculated based on Eq. (4) and stabilisation factor (*S;* Eq. (5)) were calculated for each plot, according to Keuskamp et al. (2013) (Eqs. (4), (5)).

(4) $$W\left(t\right)={\mathrm{ae}}^{-\mathrm{kt}}+\left(1-a\right)$$

where W(t) is the weight (g) of tea after burial for time t (days), a is the labile and 1-a is the recalcitrant fraction of the tea (see Keuskamp et al., 2013 for further explanation.

(5) $$S=1-\frac{a_{g}}{H_{g}}$$

where *a*_*g*_ is the decomposable fraction and *H*_*g*_ is the hydrolysable fraction of green tea.

The % mass loss of Lipton roobois tea (Lipton®) and a roobois tea readily available in Colombia after 30 days was also compared to determine whether the Colombian tea brand could in future be used as a substitute for the Lipton tea.

### 2.5 Statistical analyses

Initially the data from soil samples collected adjacent to and >10 cm from a plant in the BhMulato and Pmax plots were compared to identify whether position relative to the plant had a significant effect on any of the measured soil properties. The analyses were performed using the general ANOVA tool for each forage to carry out a 2-way ANOVA with position*year as the treatment structure and block/plot as the block structure, a p value ≤ 0.05 was considered significant. Subsequently the data for each soil property obtained from the two positions (adjacent to and far from a plant) for the *Pmax* and *BhMulato* plots were combined and weighted by multiplying the mean values for samples collected adjacent to grass tufts in each plot by the proportion of ground covered by grass (*Pmax* = 0.628; *BhMulato* = 0.552) and the mean value for samples collected from >10 cm from a grass tuft by the proportion of bare ground, (Pmax = 0.372; *BhMulato* = 0.448), these weighted values were added together to give an estimate of the value for the whole plot. The estimated whole plot values for the four planted plots (*Pmax, BhMulato*, *Bh679*, *Bh16888*) were then compared with those for the bare soil control plots using the general ANOVA tool, when analyses were carried out in 2016 and 2017 the following treatment structure was used (planted /forage)*year, where ‘/’ indicates a nested design and ‘*’ a factorial design. Data from a single year were analysed with the nested structure planted/forage, in all cases the blocking structure block/plot was used. Due to a non-normal distribution, data for total soil sugar and hexose: pentose sugar ratios were transformed by log_10_ to produce a data set with a normal distribution on which to perform the ANOVA. A p value ≤ 0.05 was considered significant, where a significant effect of forage variety is identified and there is no interaction with sampling year, the least significant difference (L.S.D) for the forage means averaged across both years is given, where there is an interaction with year the L.S.D. for forage variety within year is given.

Results from the Teabag test (*k and S*) were analysed using the general ANOVA tool with planted/ forage as the treatment structure and block/plot as the block structure, a p value ≤ 0.05 was considered significant.

Using the data from soil samples collected in 2017, correlations (Pearson correlation coefficient; r) were determined between the variables SOC concentration, total soil sugar concentration (Tot_sugar), total n-alkane concentration (Tot_alkane), SoilLoss and F. The significance for the correlation was determined using a two-tailed *t*-test, a p value ≤ 0.05 was considered significant. Subsequently the contributions of the proposed explanatory variables; i) SOC concentration, ii) Tot_sugar, and iii) Tot_alkane to the response variables; a) SoilLoss and b) F, was determined using a general linearised regression to identify the necessary terms for a model predicting each response variate. All analyses were performed using GENSTAT 18 software.

## 3 Results

### 3.1 Effect of position relative to plant on soil properties in BhMulato and Pmax plots

SOC concentrations were significantly greater next to the plant than further from the plant under BhMulato, but the difference was not significant for Pmax (Table 1). Total soil sugars and total soil n-alkanes did not differ significantly between soils taken from adjacent to or >10 cm from the plant under either BhMulato or Pmax. The Hexose:Pentose sugar ratio was significantly greater in soil samples collected >10 cm from the plant compared to adjacent to the plant under Pmax, there was no significant effect of sample position on soil Hexose:Pentose ratio under BhMulato.

**Table 1:** Mean (n = 3) soil organic carbon (SOC) concentration (% dry mass), soil loss through 50 μm mesh on wet sieving (% dry mass), Friability (F statistic), soil sugar concentrations (mg g^−1^ dry soil; glucose, galactose, mannose, arabinose, xylose, and rhamnose), hexose: pentose (mannose + galactose: arabinose + xylose) ratio, total soil N-alkane concentrations (C20-C33; ng g^−1^ dry soil), and gene copy numbers (copies mg^−1^ dry soil) of fungal 18S, bacterial 16S and ammonia monooxygenase (amoA) genes of soil samples collected to 10 cm depth from field plots of *Panicum maximum* (Pmax) and Brachiaria hybrid cv Mulato (BhMulato) in 2016 and 2017, samples were collected from either next to, or > 10 cm from the base of a plant. Values in brackets show 1 standard deviation. Results (F statistic and p value) for the effect of sample position on measured value determined by general ANOVA with position*year or position as the treatment structure and block as the block structure, p value ≤ 0.05 was considered significant.

Soil property		Pmax 2016	Pmax 2017	BhMulato 2016	BhMulato 2017
SOC	Next to plant	1.52 (0.22)	1.59 (0.17)	1.79 (0.02)	1.72 (0.05)
	> 10 cm from plant	1.31 (0.22)	1.58 (0.14)	1.55 (0.18)	1.53 (0.09)
	F (1,18)	0.9		12.12	
	P	0.379		0.013	
Soil loss on wet sieving	Next to plant	0.95 (0.14)	4.39 (0.31)	1.22 (0.30)	4.3 (0.48)
	> 10 cm from plant	1.11 (0.21)	5.45 (0.37)	1.30 (0.33)	4.9 (0.44)
	F (1,18)	70.59		15.58	
	P	<0.001		<0.001	
Friability	Next to plant	NA	0.26 (0.006)	NA	0.25 (0.03)
	> 10 cm from plant	NA	0.20 (0.01)	NA	0.21 (0.0008)
	F (1,2)	NA	39.61	NA	3.54
	P	NA	0.024	NA	0.201
Total soil sugar concentration	Next to plant	569.2 (527.9)	220.5 (44.52)	398. (279.9)	156.2 (42.3)
	> 10 cm from plant	272.2 (115.4)	172.1 (58.2)	369.4 (154.2)	178.5 (9.25)
	F (1,18)	1.43		0	
	P	0.277		0.977	
Hexose:Pentose	Next to plant	0.18 (0.07)	0.32 (0.06)	0.24 (0.027)	0.35 (0.026)
	> 10 cm from plant	0.31 (0.04)	0.37 (0.008)	0.21 (0.09)	0.37 (0.005)
	F(1,18)	9.26		0.1	
	P	0.023		0.767	
Total soil alkane concentration	Next to plant	NA	2.51 (1.03)	NA	3.84 (1.96)
	> 10 cm from plant	NA	1.43 (0.26)	NA	1.92 (0.13)
	F (1,2)	NA	2.77	NA	2.54
	P	NA	0.238	NA	0.252
Fungal 18S	Next to plant	7.92 × 10^4^ (4.69 × 10^4^)	NA	11.13 × 10^4^ (12.84 × 10^4^)	NA
	> 10 cm from plant	5.79 × 10^4^ (2.78 × 10^4^)	NA	5.57 × 10^4^ (2.62 × 10^4^)	NA
	F (1,2)	0.26	NA	0.89	NA
	p	0.664	NA	0.446	NA
Bacterial 16S	Next to plant	0.80 × 10^7^ (0.33 × 107)	NA	1.46 × 10^7^ (0.46 × 10^7^)	NA
	> 10 cm from plant	1.21 × 10^7^ (0.71 × 10^7^)	NA	2.05 × 10^7^ (0.59 × 10^7^)	NA
	F (1,2)	0.92	NA	12.61	NA
	p	0.438	NA	0.071	NA
AMOA	Next to plant	0.26 × 10^4^ (0.11 × 10^4^)	NA	0.66 × 10^4^ (0.21 × 10^4^)	NA
	> 10 cm from plant	1.87 × 10^4^ (2.52 × 10^4^)	NA	1.37 × 10^4^ (0.47 × 10^4^)	NA
	F (1,2)	1.13	NA	0.457	NA
	p	0.399	NA	0.166	NA

Under both Pmax and BhMulato the soil samples collected from adjacent to the plants showed significantly greater aggregate stability (less soil loss on wet sieving) than the samples collected from >10 cm from the plants.

Soil friability (measured in 2017 only) under Pmax was significantly greater in the samples collected from adjacent to the plant than in soils collected >10 cm from the plant, there was no significant effect of sample position on soil friability under BhMulato.

The concentrations of key microbial marker genes (measured in 2016 only), including fungal 18S bacterial 16S and amoaA genes was not significantly affected by sampling position relative to the plant in either the Pmax of BhMulato plots.

### 3.2 Comparison of soil properties between forages

**Fig. 3:**
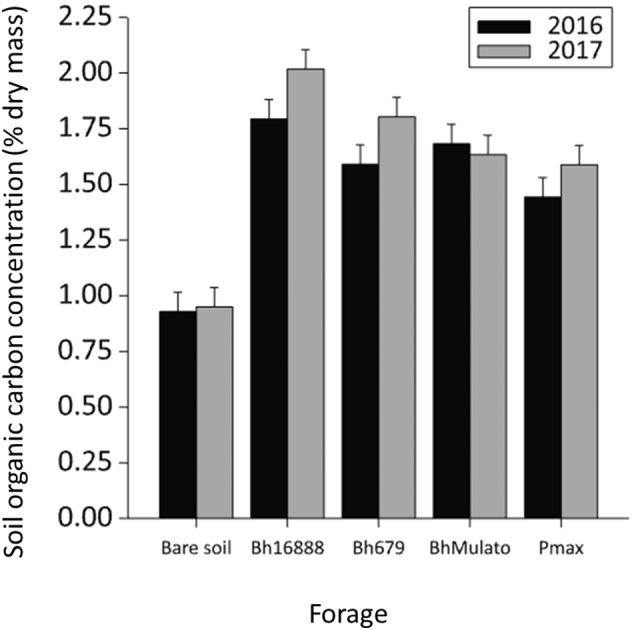
Mean (n = 3) soil organic carbon concentration (% dry mass) for samples collected under four forage varieties (*Brachiaria hybrid cv* Mulato (BhMulato)*, B.humidicola cv* Tully (CIAT679; Bh679*), B.humidicola cv CIAT16888 (*Bh16888*),* and *Panicum maximum (*Pmax) and a bare soil control in 2016 and 2017. Error bars show 1 standard error of the mean.

The presence / absence of plants had a significant effect on SOC concentration (F(1,17) = 120.30, p < 0.001) with bare soil having significantly less SOC than the planted plots (Fig. 3). There was also a significant effect of forage variety (F(3,117) = 6.87, p = 0.003) on SOC concentration, but no significant effect of sampling year (F(1,17) = 4.08, p = 0.059). Averaged across both years Bh16888 had the greatest mean SOC concentrations followed by Bh679, BhMulato and Pmax (1.91, 1.70, 1.66, 1.52% dry mass respectively; L.S.D. = 0.18).

**Fig. 4:**
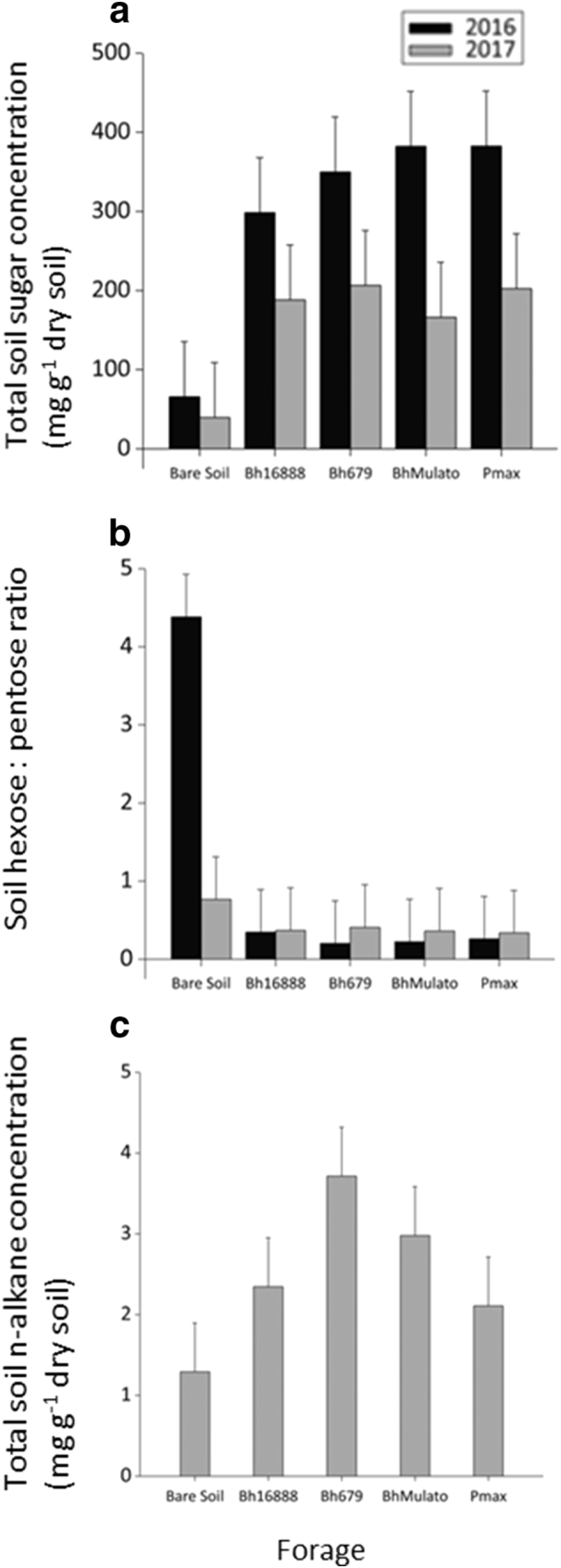
Mean (n = 3) a) total soil sugar concentration (mg g^−1^ dry soil; sum of glucose, galactose, mannose, arabinose, xylose, rhamnose) b) hexose: pentose ratio (galactose + mannose: arabinose: xylose) and c) total soil n-alkane concentration (ng g^−1^ dry soil) for samples collected from under four forage varieties (*Brachiaria hybrid cv* Mulato (BhMulato)*, B.humidicola cv* Tully (CIAT679; Bh679*), B.humidicola cv CIAT16888 (*Bh16888*),* and *Panicum maximum (*Pmax) and a bare soil control in 2016 and 2017. Error bars show 1 standard deviation.

The bare soil samples had significantly lower total sugar concentrations compared to the planted plots (F(1,8) = 68.98, p < 0.001; Fig. 4), but there was no significant difference in total soil sugar concentration between the forage varieties (F(3,8) = 0.10, p = 0.957). The soils sampled in 2016 had a greater soil sugar concentration than those sampled in 2017 (F(1,10) = 13.05, p = 0.005) The hexose:pentose sugar ratio was significantly greater in the bare soil compared to the soil under the forages (F(1,8) = 117.22, p < 0.001;) but there was an interaction effect with the sampling year (F(1,9) = 27.26, p = 0.001). There was no significant difference in the hexose: pentose sugar ratio under the four different forage varieties (F(3,8) = 0.30, p = 0.828).

There was no significant difference at the 5% confidence level in total soil n-alkane concentration between the bare soil and planted plots (F (1,8) =4.88, p = 0.058) although the bare soils tended to have lower total soil n-alkane concentrations (Fig. 4). There was no significant difference between the forage varieties in total soil n-alkane concentration (F (3,8) =1.41, p = 0.309; Fig. 4).

**Fig. 5:**
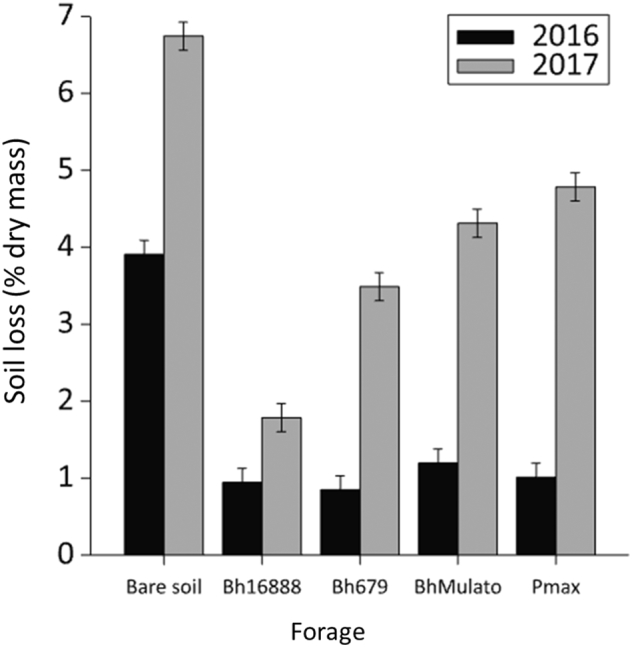
Mean (n = 3) % soil lost through 50 μm mesh on wet sieving (% dry soil mass) for samples collected under four forage varieties (*Brachiaria hybrid cv* Mulato (BhMulato)*, B.humidicola cv* Tully (CIAT679; Bh679*), B.humidicola cv CIAT16888 (*Bh16888*),* and *Panicum maximum (*Pmax) and a bare soil control in a) 2016 and b) 2017. Error bars show 1 standard error of the mean.

The bare soil had significantly less stable aggregates (greater soil loss on wet sieving) than the planted soils in both years (F(2,8) = 403.16; p < 0.001; Fig. 5). There was a significant difference in soil aggregate stability between the forage varieties (F(3,8) = 26.61; p < 0.001) and between the two sample years (F(1,10) = 569.40; p < 0.001); all soils sampled had much greater aggregate stability in 2016. There was a significant interaction effect between the sampling year and forage variety on soil aggregate stability (F(3,10) = 25.82; p < 0.001) the BhMulato (1.20 and 4.31% soil loss in 2016 and 2017 respectively) and Pmax (1.01 and 4.78% soil loss in 2016 and 2017 respectively) plots had less stable soil aggregates than the Bh16888 (0.95 and 1.79% soil loss in 2016 and 2017 respectively) plots in both years. In 2016 the soils sampled from under Bh679 (0.85% soil loss) showed a similar aggregate stability to those under Bh16888, whereas in 2017 the soil aggregates under Bh679 (3.49% soil loss; L.S.D. = 0.54) were found to be less stable (greater soil loss) than those under Bh16888.

**Fig. 6:**
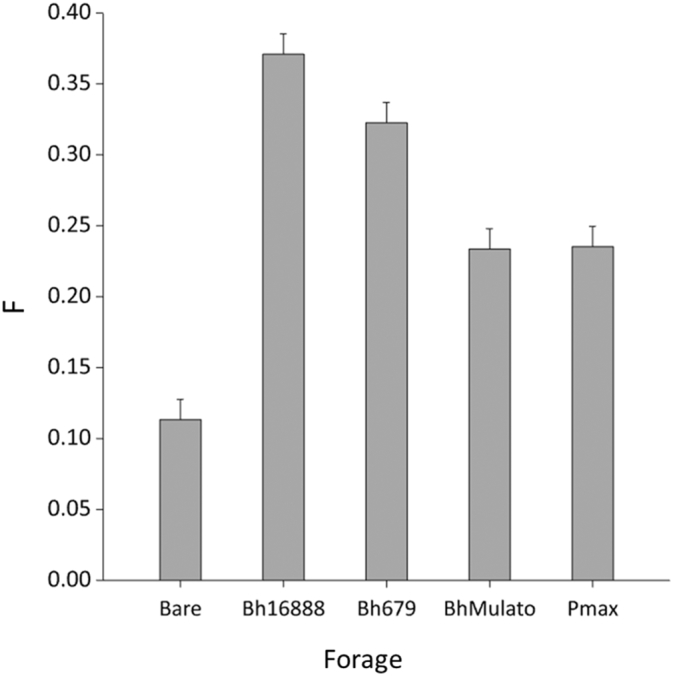
Mean (n = 3) soil Friability (F statistic) for samples collected under four forage varieties (*Brachiaria hybrid cv* Mulato (BhMulato)*, B.humidicola cv* Tully (CIAT679; Bh679*), B.humidicola cv CIAT16888 (*Bh16888*),* and *Panicum maximum (*Pmax) and a bare soil control in 2017. Error bars show 1 standard error of the mean.

The bare soil was found to be significantly less friable than the soils planted with forages (F(1,8) = 122.71; p < 0.001; Fig. 6). There were significant differences in soil friability between the four forage varieties (F(3,8) = 22.45; p < 0.001) with the most friable soils being observed under Bh16888 (0.3709 F statistic) followed by Bh679 (0.3226 F statistic). The soils under Pmax and BhMulato showed similar levels of friability (0.2353 and 0.2334 F statistics respectively; L.S.D. = 0.05).

There was a significant negative correlation between SoilLoss (proportion soil loss on wet sieving) and SOC concentration (Table 2) as well as between SoilLoss and Tot_sugars the correlation between SoilLoss and Tot_alkanes (total soil alkane concentration) was insignificant, indicating that WSA (inversely proportional to SoilLoss) increased with increasing SOC and Tot_sugars but was less influenced by Tot_alkanes. Soil Friability (F) was significantly positively correlated with SOC, and Tot_sugars but not significantly correlated with Tot_alkanes. There was a positive correlation between SOC and Tot_sugars and SOC and Tot_alkanes.

**Table 2:** Pearson correlation coefficient and significance (p value; in brackets) from a two-tailed *t*-test for pairwise combinations of soil variables including soil organic carbon concentration (SOC), soil loss on wet sieving (SoilLoss), total soil sugar concentration (Tot_sugar), total soil alkane concentration (Tot_alkane) and Friability (F statistic; F).

	SoilLoss	SOC	Tot_sugar	Tot_alkane	F
SOC	−0.851 (<0.001)				
Tot_sugar	−0.555 (0.0091)	0.663 (0.0014)			
Tot_alkane	−0.416 (0.0611)	0.483 (0.0311)	0.2248 (0.3273)		
F	−0.9463 (<0.001)	0.888 (0.001)	0.635 (0.002)	0.404 (0.0696	

Regression analysis to obtain the optimal model for the response variable SoilLoss identified that from a maximal model containing the proposed explanatory variables SOC + Tot_sugar+ Tot_alkane, the SOC term was significant (t(16) = −4.51, p < 0.001) whilst the Tot_sugar (t(16) = 0.22 p = 0.825) and Tot_alkane (t(16) = 0.27, p = 0.789)) terms were insignificant. Sequential removal of terms revealed the optimal linear model to describe Soil loss was:

−3.646×SOC+10.313=Soil loss.

The model fit was highly significant (F(1,18) = 47.4 p < 0.001), it explained 70.9% of the variance in SoilLoss, with a residual mean square of 0.6732.

Regression analysis to obtain the optimal model for the response variable F identified that from a maximal model containing the proposed explanatory variables SOC + Tot. sugar+ Tot. n-alkane, the SOC term (t(16) = 5.01, p < 0.001) was significant and the Tot. sugars (p = 0.684) and Tot. n-alkanes terms were insignificant (p = 0.757). Sequential removal of terms revealed the optimal linear model to describe F was:

0.2111×SOC−0.00920=F

The model fit was highly significant (F(1,18) = 67.31, p < 0.001), it explained 77.7% of the variance in F with a residual mean square of 0.001588.

**Fig. 7:**
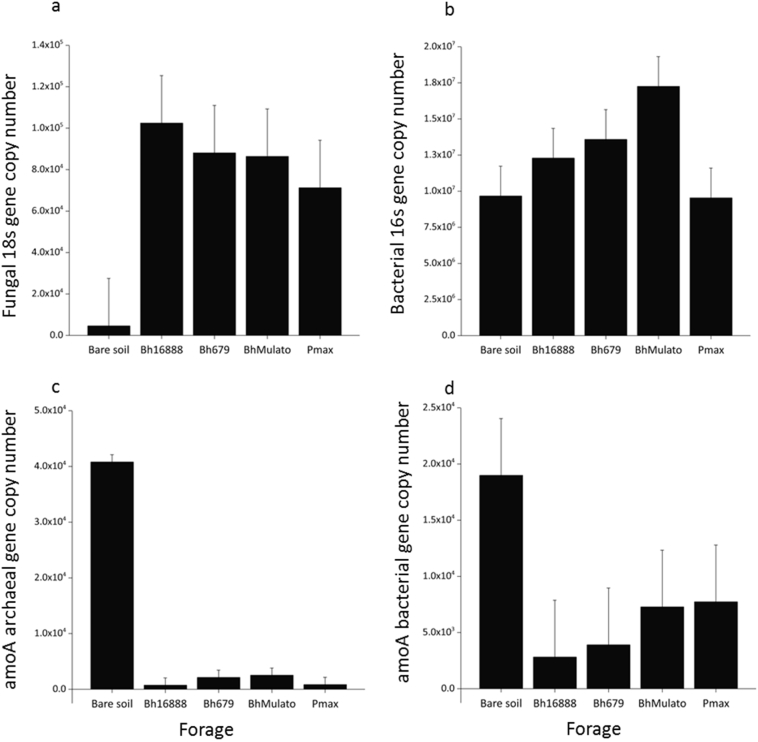
Mean (n = 3) gene copy number (copies mg^−1^ dry soil) in soil samples collected under four forage varieties (*Brachiaria hybrid cv* Mulato (BhMulato)*, B.humidicola cv* Tully (CIAT679; Bh679*), B.humidicola cv CIAT16888 (*Bh16888*),* and *Panicum maximum (*Pmax) and a bare soil control in 2016, showing results for a) fungal 18S gene, b) bacterial 16S gene, c) total ammonia monooxygenase A (amoaA) archaeal genes, d) total amoaA bacterial genes. Error bars show 1 standard error of the mean.

The fungal 18S gene copy number was significantly lower in the bare soil samples compared to the planted soils (F(1,8) = 10.37, p = 0.012; Fig. 7), but there was no significant difference between the forages (F(3,8) = 0.31, p = 0.817). The bacterial 16S gene copy number did not differ significantly between the bare soil and planted plots (F(1,8) = 2.31; p = 0.167) or between the different forage varieties F(3,8) = 2.42, p = 0.141). The bare soil had significantly greater amoA archaeal genes (F(1,8) = 725.52, p < 0.001) and significantly greater amoA bacterial genes (F(1,8) = 5.76, p = 0.043) but there was no significant difference in amoA archaeal (F(3,8) = 0.48, p = 0.706) or amoA bacterial (F(3,8) = 0.23, p = 0.871) gene copy number between the forage varieties.

**Fig. 8:**
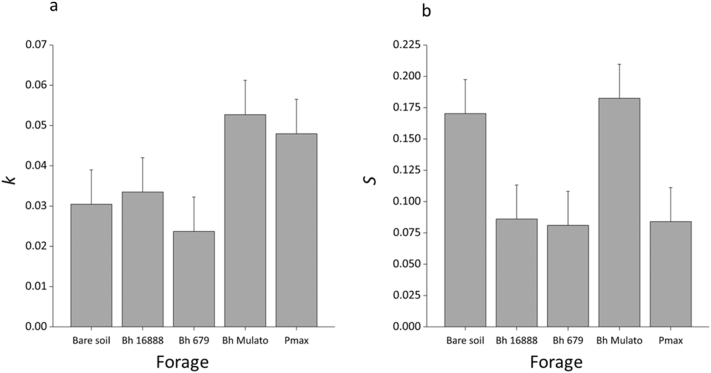
Mean (n = 3) litter deomposition rate (*k; a*) and stabilisation factor (*S; b*) calculated from teabags buried for 30 days under forage varieties (*Brachiaria hybrid cv* Mulato (BhMulato)*, B.humidicola cv* Tully (CIAT679; Bh679*), B.humidicola cv CIAT16888 (*Bh16888*),* and *Panicum maximum (*Pmax) and a bare soil control in 2017. Error bars show standard error.

ANOVA revealed no significant effect of the presence or absence of forage grass on the decomposition rate of the tea litter (*k*; F (1,8) = 0.89; p = 0.373; Fig. 8) or the stabilisation factor (*S*; F(1,8) = 4.16, p = 0.076). Likewise the different forage varieties had no significant effect on *k* (F(1,3) = 2.43, p = 0.140) of *S* (F(1,3) = 3.32, p = 0.078).

**Fig. 9:**
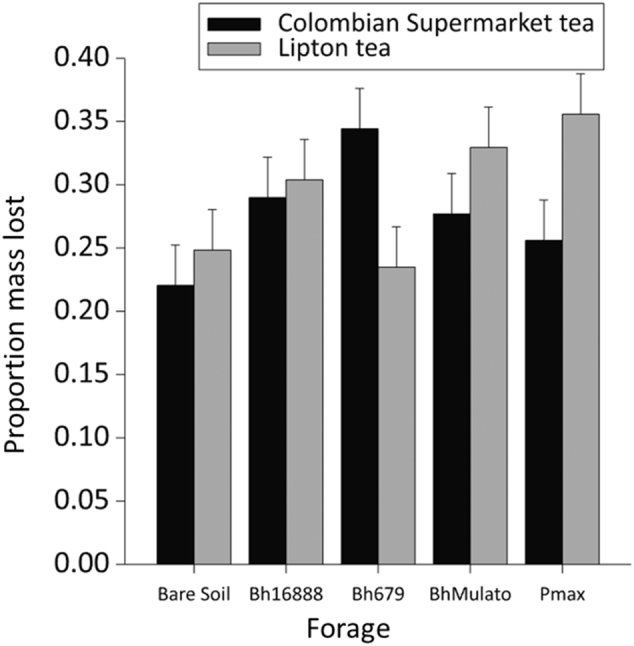
Mean (n = 3) percentage mass loss of roobois tea leaves from teabags buried for 30 days under forage varieties (*Brachiaria hybrid cv* Mulato (BhMulato)*, B.humidicola cv* Tully (CIAT679; Bh679*), B.humidicola cv CIAT16888 (*Bh16888*),* and *Panicum maximum (*Pmax) and a bare soil control in 2017. Tea bags were sourced either from a Colombian supermarket or were Lipton© teabags sourced from the Netherlands according to the method of Keuskamp et al. (2013). Error bars show 1 standard deviation.

Comparison of the proportion of total tea mass lost between the two brands of roobois tea revealed a significant interaction effect between the brand of tea and forage variety (F(4,20) = 3.03, p = 0.042; Fig. 9).

## 4 Discussion

SOC concentration is a key determinant of soil health; increased SOC concentrations improve soil structure, support a more diverse micro and macro fauna and provide a regulated supply of nutrients to plants (Doran and Zeiss, 2000). The significantly lower SOC concentration in the bare soil plots in this study can be explained by the absence of fresh inputs from plant matter and continued decomposition of remaining SOC by soil microbes. We also identified significant differences in SOC concentration between soils under the four forage varieties, evidence that differences in forage properties could be driving differences in SOC accumulation. Previous studies have identified significant variation amongst tropical forage varieties in properties such as; growth rate, relative allocation of carbon to different plant tissues (e.g. above ground vs below ground allocation), litter composition (e.g. C:N ratio) and the quantity / composition of plant root exudates (Dubeux et al., 2007), all of which have been shown to impact SOC concentrations (Kögel-Knaber, 2002). Reduced input of organic matter e.g. from root turnover, root exudates and leaf litter has been shown to result in lower soil organic matter concentrations in the gaps between tussock forming grasses compared to directly under tussocks (Hook et al., 1991; Gregory et al., 2018). In our study SOC concentrations were significantly greater next to the plants than in the gaps between tussocks in the BhMulato plots but not the Pmax plots.

As well as measuring bulk SOC concentration we also compared organic matter composition under the different forage varieties by measuring the concentrations of sugars and n-alkanes. Sugars are the most abundant group of organic molecules, they provide a readily available energy source to soil microbes, are the major component of plant root exudates and have been suggested as playing a role in the formation of water stable aggregates (Gunina and Kuzyakov, 2015). The results from our study show high inter plot variability in total soil sugar concentration with no significant differences between the forage varieties, so provide no evidence of a difference in the quantity of root-exudates from the different grass varieties. The ratio of hexose:pentose sugars can be used to infer the dominant source of sugars in soils, as microbes predominantly synthesise galactose and mannose (hexose sugars), whereas plants synthesise both hexose and the pentose sugars arabinose and xylose (Oades, 1984). The lack of a significant effect of forage variety on the soil hexose:pentose sugar ratio in this study, suggests the relative input of plant vs microbially derived organic matter is similar for all varieties, whilst the greater hexose:pentose ratio in the gaps between Pmax tussocks and in the bare soil plots and the reduced total soil sugar concentrations in the bare soil likely reflect the reduced input of fresh plant material.

N-alkanes are another group of organic compounds that can form a major component of free soil lipids in grassland systems (Otto and Simpson, 2005) and play an important role in soil function; they are highly non-polar and therefore hydrophobic so can prevent water entering soil aggregates, thus increasing aggregate stability (Piccolo and Mbagwu, 1999). Once in the soil, n-alkanes can accumulate and, as they are a component of plant epicuticular waxes, form a record of plant material input over many decades (Bush and McInerney, 2013). The lack of significant differences in soil n-alkane concentration under the different treatments in this study suggests that rates of plant matter input over the mid to long-term have been similar under all the forage varieties. Even under the bare soil plots the n-alkane concentration was not significantly lower, which likely reflects the relatively slow rate of decomposition of n-alkanes accumulated under the previous land use, emphasising that a longer time may be required before any differences in n-alkane input under the different forages have a significant effect on soil concentrations.

Changes in quantity and composition of organic matter input to soil are known to have short and longer-term effects on aggregate stability (Abiven et al., 2009), as organic compounds play a key role in preventing water from entering aggregates through hydrophobicity and enhancing aggregate cohesion (Chenu et al., 2000). Significant increases in aggregate stability have been found under no tillage compared to conventional tillage and in permanent pasture compared to cultivated arable soils (Beare et al., 1994; Douglas and Goss, 1987). To our knowledge few studies have investigated differences in stability due to forage variety, particularly in the tropics. Our study found significantly less stable aggregates in the bare soil plots (greater percentage soil mass loss on wet sieving) compared to the forage plots, whilst soils under Bh679 and Bh16888 were found to have more stable aggregates than the soils from the BhMulato and Pmax plots. The improved aggregate stability under Bh679 and Bh16888 is likely to make the soils less vulnerable to erosion (Le Bissonais, 1996).

As with aggregate stability, friability has been shown to be affected by tillage, and organic matter concentration, tending to increase as SOC increases and decrease in response to tillage (Watts and Dexter, 1998). The correlation between SOC and friability in our study demonstrates how differences in SOC could be driving the significant differences in soil friability observed. The greater soil friability could explain the observation that soil sampling under the Bh679 and Bh16888 plots was much easier than sampling in the Pmax and BhMulato plots (L. A. Lopez, Field Team Leader, CIAT, 2016, personal communication, January), as soil that is more friable is easier to till (Watts and Dexter, 1998), this is an important consideration, particularly for farmers in developing nations in the tropics where soil cultivation is carried out manually. The greater soil friability under Bh679 and Bh1688 could also enable root growth and allow for more efficient water and nutrient uptake (Passioura, 1991), thus improving drought tolerance and forage nutritional quality.

Spatial variation in SOC concentrations under tussock forming grasses (observed under BhMulato but not Pmax in our study plots) could be expected to lead to corresponding differences in aggregate stability and friability. For both the tussock forming grass species in our study (Pmax and BhMulato), the soils taken from adjacent to the tussocks were significantly more stable than those taken from the gaps between the tussocks, whilst soil friability was significantly affected by sample position in the Pmax plots but not in the BhMulato plots. The inconsistent impact of sampling position on SOC concentration, aggregate stability and friability in our study suggests that, whilst SOC concentration plays a role in regulating aggregate stability and friability, the direct impact of root growth, which concentrates beneath tussocks, may also be having a significant effect on the spatial variation in soil physical properties observed. This finding is similar to that made by Imhoff et al. (2000) when studying the tussock forming grass *Pennisetum purpureum.*

N-alkanes and sugars have been proposed as playing a key role in the soil physical properties of aggregate stability and friability, which have also been found to be positively correlated with total SOC concentrations (Abiven et al., 2009; Watts and Dexter, 1998). We assessed which of these potential explanatory variables best explained the differences observed in soil physical properties in this study, all three were positively correlated with one another and with both aggregate stability and friability. Regression analyses revealed that SOC concentration alone best described the variation between samples. Our data provide no evidence for sugars or alkanes having a specific role in regulating friability or aggregate stability, supporting the findings of others who have found no specific correlation between total sugar concentration (Baldock et al., 1987) and aggregate stability. Further work on the differences in organic matter composition between the plots in this study may help to reveal whether any other group of organic compounds has a specific role to play in aggregate stabilisation in the plots.

We used qPCR to compare the abundance of genes from the major microbial groups in the soils of our study plots. Plant species identity can drive significant differences in soil microbial communities (Bezemer et al., 2006) due to differences in composition and quality of litter inputs, rates of nutrient uptake and production of root exudates. For example archaea abundance tends to increase with decreasing C:N ratio, whilst higher levels of inorganic N availability can increase the competitive advantage of bacteria over archaea (Bates et al., 2010). In turn differences in microbial community composition can be important drivers for soil health. For example, fungal dominated communities tend to be associated with greater rates of organic matter accumulation (Six et al., 2006), slower rates of nutrient cycling (van der Heijden et al., 2008) and reduced N losses via leaching and gaseous emissions (De Vries et al., 2011). Increased fungal biomass can also increase the formation of stable macroaggregates (Bossuyt et al., 2001; Rillig et al., 2002). We assessed the abundance of amoA genes; important functional genes involved in the N-cycle. Soils with reduced amoA gene abundance, particularly amoaA archaeal genes (Byrnes et al., 2017; Subbarao et al., 2009), have been found in the rhizosphere of forage varieties exhibiting high BNI activity (Nuñez et al., 2018), due to the production of allelopathic compounds (Moreta et al., 2014). Of the species in our study, Bh679 and Bh16888 exhibit higher levels of BNI activity compared to BhMulato and Pmax (Nuñez et al., 2018). Our results showed significantly lower 18S gene fungal abundance and greater amoA gene abundance in the bare soils compared to the planted soils, but no significant effect of forage variety, or position relative to the plant in the Pmax and BhMulato plots, on the abundance of any of the marker genes quantified. Prolonged absence of plant matter input to the soil and absence of living roots for plant fungal symbiosis (Eom et al., 2000) can explain the significantly different microbial communities in the bare soil. The lack of a significant effect of forage variety on marker gene abundance in our study plots could indicate limited differences in the abundances and activity of the corresponding microbial groups. Alternatively, this could be due to the limitations of using the quantification of DNA to assess soil microbial communities, as this does not provide information on which genes are being actively transcribed. Further analysis of mRNA content of the soils could reveal differences in microbial activity under the different forages (Urich et al., 2008), which has been found to correlate more with soil properties than microbial abundance (De Gryze et al., 2005).

To compare the activity of the microbial communities in our plots, we measured litter decomposition rates using the teabag test. Organic matter decomposition is a vital process for many soil functions (Kibblewhite et al., 2007). Greater rates of decomposition indicate a more active microbial community that can drive faster rates of nutrient recycling. A more active soil microbial community has been suggested as an indicator of improved soil health (Enriqueta Arias et al., 2005). Differences in the composition and activity of the soil microbial community can be driven by differences in management and have been shown in some cases to affect the rate of litter decomposition (Beare et al., 1992; Keeler et al., 2009; Milcu et al., 2011). We carried out the ‘teabag test’ (Keuskamp et al., 2013) to identify any differences in the rate of microbe driven litter decomposition between the plots. The test uses a common litter to compare the relative activities of the microbial communities, since rates of litter decomposition (Silver and Miya, 2001) and stabilisation (Berg and Meetenmeyer, 2002) can also be affected by litter composition. All the soils in our study had very similar stabilisation and decomposition factors, even where there were significant differences in microbial marker gene concentrations, as was found between the bare soils and planted plots. Our results show that the microbial communities present in all the study plots have similar potential to decompose a common litter and support the hypothesis that climatic differences have the greatest impact on decomposition potential of a common litter (Keuskamp et al., 2013).The observed trends for differences in SOC accumulation between our plots are likely to be a direct result of differences in inputs rather than differences in microbial communities a hypothesis supported by the observation that using the alternative roobois tea brand did have a significant effect on measured decomposition rates.

The soil properties found to show the greatest variation between forage varieties in this study were, aggregate stability, friability and SOC concentration, these three soil properties were also found to be highly correlated with one another. It is likely that variations in plant traits play a significant role in regulating these key indicators of soil health. The forage varieties included in this study show high levels of morphological and physiological diversity. Identifying which of these diverse plant traits have the potential to improve soil health could aid the future breeding of forage varieties that, along with having favourable agronomic traits such as high productivity and disease and drought resistance, also support the development of healthy soil. Plant traits that have been found to have a positive effect on aggregate stability include increased total root length (Haynes and Francs, 1993) and a consistent root distribution throughout the soil (Roberson and Firestone, 1991). Previous work carried out in 2013 and 2014 at CIAT found BhMulato to have a consistently smaller total root length compared to the other forage varieties in our study, whilst in the second year of sampling (2014) Bh16888 was found to have the greatest total root length (unpublished; see Supplementary Table 2). This difference in root production could partially explain the lower aggregate stability found under BhMulato compared to under Bh679 and 16,888. Under Pmax the total root length in the 0–10 cm depth range was found to be very variable between years, which could lead to corresponding high inter-annual variability in aggregate stability.

Plant productivity is another property likely to influence, SOC concentration, aggregate stability and friability, increased inputs of plant litter to soil can support aggregate formation and stabilisation (De Gryze et al., 2005). Previous work on the study plots compared dry matter yield between July 2010 and August 2015 and revealed significant interactions between sampling date and forage variety on yields, but no single forage variety was consistently more or less productive than the others, (unpublished, see Supplementary Fig. 1). Suggesting that dry matter yield alone may not be the best predictor for SOC concentrations and that other considerations such as litter quality could also be important in influencing the impact of the different forage varieties on SOC stocks (Angers and Caron, 1998).

Inter-annual variations in forage yield and root growth, like those observed in the previous studies at our field plots, are likely to impact on the soil health indicators we measured. Weather conditions are a key contributor to such variations in growth. During the 2016 sampling our study site was experiencing extreme drought (The Bogota Post, 2016), whereas during our 2017 sampling rainfall levels were more typical for the region. This could explain the greater aggregate stability we observed in 2016, as drought conditions could have encouraged increased plant root growth to maximises water uptake, thus increasing aggregate stabilisation. Further research is needed to understand the interaction between climate and forage variety on soil health in the tropics.

This study has demonstrated the potential to improve soil physical properties and increase SOC storage by planting specific *Brachiaria* cultivars, in particular Bh16888 and Bh679 were shown to have the most positive influence on soil health, with the soils under these cultivars having the greatest aggregate stability, being the most friable and having the highest SOC concentration, when compared to Pmax and BhMulato, particularly in the second year of the study. Previous studies on these tropical forages have shown that Bh16888 and Bh679 also benefit from having high BNI potential, with reduced nitrification rates in soil, (Nuñez et al., 2018) and soils under Bh679 show reduced emissions of the greenhouse gas Nitrous oxide (N_2_O; Byrnes et al., 2017).

## 5 Conclusion

With this study we aimed to compare soil health under different tropical forage varieties to identify those with the potential to improve agricultural sustainability. Significant differences in aggregate stability, friability and SOC concentrations all suggest potential for improved soil health under Bh679 and Bh16888. Our findings of significant improvement in soil health indicators adjacent to tussocks, as compared to in gaps between tussocks highlight the role of growth habit in driving spatial variation in soil health. We suggest that the impacts of potential new commercial forage cultivars on soil health and function should be a key property of consideration for future breeding programmes and be one of the traits considered by land managers when selecting which varieties to use.

We measured multiple indicators to identify the most suitable low and high-tech method to assess soil health in the field or lab and identify the relationships between these indicators. Total SOC concentrations were strongly correlated with aggregate stability and friability, and both aggregate stability and friability could be easily measured at low or no cost without the need for high-tech equipment. Hence important improvements in soil health could be made and monitored throughout the tropics by smart forage selection and the use of low tech methods.

## Declaration of Competing Interest

None.

## Appendix A Supplementary data

Supplementary material Image 1
