# Supplementary material for: Smart forage selection could significantly improve soil health in the tropics
Source: Sci Total Environ. 2019 Oct 20;688:609–21. doi: 10.1016/j.scitotenv.2019.06.152 (PMC6708042; doi:10.1016/j.scitotenv.2019.06.152)
Supplement: Supplementary file 1 — Supplementary material [file mmc1.docx]

Supplementary data

**Supplementary Table 1** Table showing % surface cover by grass in each of five randomly located 1 m^2^ quadrats in January 2017, for three replicate plots of *Brachiaria hybrid cv* Mulato (BhMulato) and *Panicum maximum* (Pmax) grasses.

| Block | Quadrat | % grass cover Bh Mulato | % grass cover Pmax |
| --- | --- | --- | --- |
| 1 | 1 | 48 | 66 |
|  | 2 | 67 | 50 |
|  | 3 | 64 | 75 |
|  | 4 | 47 | 70 |
|  | 5 | 49 | 59 |
|  | Mean | 55 | 64 |
| 2 | 1 | 45 | 56 |
|  | 2 | 55 | 60 |
|  | 3 | 49 | 60 |
|  | 4 | 65 | 50 |
|  | 5 | 54 | 75 |
|  | Mean | 53.6 | 60.2 |
| 3 | 1 | 60 | 70 |
|  | 2 | 60 | 60 |
|  | 3 | 63 | 55 |
|  | 4 | 55 | 70 |
|  | 5 | 50 | 66 |
|  | Mean | 57.6 | 64.2 |

**Supplementary Table 2** Mean (n=3) Total root length of four forage varieties: *Brachiaria hybrid cv* Mulato (BhMulato)*, B.humidicola cv* Tully (CIAT679; Bh679*), B.humidicola cv CIAT16888 (*Bh16888*),* and *Panicum maximum (*Pmax) (km root m^-2^soil), in given soil depth range (cm) as measured in 2013 and 2014 on field plots established in 2006 at CIAT in Palmira, Valle del Cauca Colombia (3⁰ 30’7’’N 76⁰21’22’’W).

|  | 2013 Total root length in given soil depth range | | | | | | | | 2014 Total root length in given soil depth range | | | | | | | |
| --- | --- | --- | --- | --- | --- | --- | --- | --- | --- | --- | --- | --- | --- | --- | --- | --- |
| Forage variety | 60-80 | 40-60 | 20-40 | 10-20 | 5-10 | 0-5 | 0-80 | 0-10 | 60-80 | 40-60 | 20-40 | 10-20 | 5-10 | 0-5 | 0-80 | 0-10 |
| BhMulato | 2.19 | 2.74 | 2.44 | 1.68 | 1.96 | 1.89 | 12.91 | 3.86 | 2.94 | 3.54 | 2.89 | 2.23 | 1.24 | 1.17 | 14.01 | 2.42 |
| Pmax | 0.85 | 0.89 | 1.68 | 2.10 | 3.37 | 9.27 | 18.16 | 12.65 | 2.46 | 1.06 | 2.00 | 4.23 | 3.88 | 4.89 | 18.51 | 8.77 |
| Bh16888 | 6.73 | 5.51 | 4.63 | 3.96 | 3.20 | 5.12 | 29.15 | 8.32 | 4.94 | 3.78 | 3.92 | 6.89 | 5.83 | 5.04 | 30.41 | 10.87 |
| Bh679 | 4.13 | 3.54 | 6.40 | 6.90 | 4.15 | 6.31 | 31.44 | 10.46 | 5.66 | 3.91 | 6.49 | 6.58 | 2.55 | 6.01 | 31.20 | 8.56 |


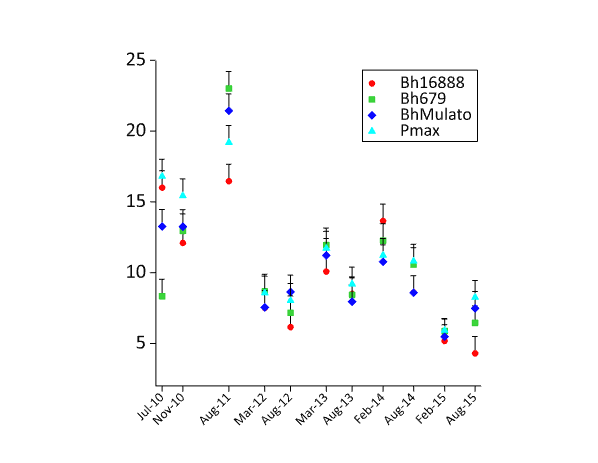


**Supplementary Figure 1**. Mean (n=3) dry matter yield (Ton dry matter ha^-1^) obtained on given harvest date (Month-Year) between July 2010 and August 2015 from field plots growing one of four forage varieties *Brachiaria hybrid cv* Mulato (BhMulato)*, B.humidicola cv* Tully (CIAT679; Bh679*), B.humidicola cv CIAT16888 (*Bh16888*),* and *Panicum maximum (*Pmax), field plots were established in 2006 at CIAT Palmira, Valle del Cauca Colombia (3⁰ 30’7’’N 76⁰21’22’’W). Error bars show 1 standard error of the mean.
